# Postoperative Pain and Matrix Metalloproteinase-9 Pulpal Blood Level in Symptomatic Pulpitis of Mature Molars Treated with Biodentine and Portland Cement Full Pulpotomy: A Randomized Clinical Trial

**DOI:** 10.4317/jced.64138

**Published:** 2026-05-29

**Authors:** Aya Ahmed Youssef-Gamal, Hany Samy Sadek, Ghada El Hilaly Mohamed Eid

**Affiliations:** 1 Master’s degree (Assistant lecturer). Department of Endodontics, Faculty of Dentistry, Cairo University, Cairo, Egypt; 2 PhD (Professor). Department of Endodontics, Faculty of Dentistry, Cairo University, Cairo, Egypt

## Abstract

**Background:**

This study compared postoperative pain after Biodentine and Portland cement full pulpotomy in mature molars with symptomatic pulpitis. Additionally, it correlated the pulpal blood level of Matrix Metalloproteinases-9 MMP-9; as a marker of the state of pulp inflammation, with the preoperative pain scores, and intra-operative bleeding time during pulpotomy.

**Materials and Methods:**

Seventy-two mature molars were randomly allocated to two full pulpotomy groups using either Biodentine or Portland cement. Intraoperatively, bleeding color, profuseness and pulp attachment were assessed under magnification of dental operating microscope. 10 µL pulpal blood sample was taken using heparinized microcapillary tube for the assessment of MMP-9 level. Hemostasis was achieved with cotton pellet soaked with 2.5% sodium hypochlorite and bleeding time was recorded. The assigned pulp capping material was placed followed by the permanent restoration. Preoperative pain scores were recorded using Numerical rating scale NRS and re-evaluated postoperatively; at 6, 12, 24, 48 and 1 week. MMP-9 concentration was measured using ELISA test. The following statistical analyses were performed to assess the data: Independent t-test, Mann Whitney test, Friedman test, Chi square test and Fisher's exact test. The significant level was set to be at P 0.05.

**Results:**

Full pulpotomy using Portland cement and Biodentine showed comparable results regarding postoperative pain at all time points. MMP-9 level showed a significant strong positive correlation with preoperative pain NRS score as well as with bleeding time. Furthermore, both preoperative scores and bleeding time revealed a significant positive correlation with postoperative pain scores at different time points.

**Conclusions:**

Full pulpotomy is an appropriate treatment option in mature molars with symptomatic pulpitis. Portland cement demonstrated promising results similar to Biodentine regarding postoperative pain relief when used as a pulp capping material in full pulpotomy. MMP-9 level in pulpal blood as well as bleeding time could offer a guiding tool similar to preoperative NRS scoring for diagnosis of pulp status. Preoperative scores and bleeding time could predict the degree of postoperative pain after full pulpotomy.

## Introduction

The advent of regenerative endodontics and the promotion of biologically based therapies have rejuvenated the application of vital pulp therapy (VPT) in mature, cariously affected teeth. Applications can target teeth with reversible as well as those with signs and symptoms indicative of irreversible pulpitis. New guidelines for VPT by the European Society of Endodontology (1) and the American Association of Endodontics (2) considered VPT as a suitable treatment for mature teeth with irreversible pulpitis considering the following: aseptic technique, sodium hypochlorite (NaOCl) for hemostasis, calcium silicate based cements as a pulp capping material, with emphasis on observation under magnification of pulp color, intensity of pulp bleeding on exposure and finally immediate permanent restoration. VPT can provide a viable treatment alternative for patients in low socio-economic communities who cannot afford root canal treatment and those in rural areas with no trained root canal treatment (RCT) providers (3). Pain relief after pulpotomy could be attributed to the decrease in the pulp chamber's local pressure and the concentration of inflammatory mediators as well as severing of terminal endings of nociceptive sensory neurons (4 , 5). Recently, long-term outcomes of coronal pulpotomy in mature permanent teeth (6 - 18) as well as short-term outcomes showed favorable results in many studies (8 , 15 , 19 - 21). In fact, full pulpotomy provided 96.49% relief from preoperative pain, as reported by Çelikkol et al. (22), with 100% relief after one week, irrespective of patient-related factors; such as age, gender, and analgesic intake. Reduction in postoperative pain after full pulpotomy as compared to RCT, was shown in some studies (4 , 8); Galani et al. (8) found a significant reduction in pain incidence in the full pulpotomy group compared to RCT. Fewer patients in the pulpotomy group reported pain (70%) compared with RCT group (100%). These results are consistent with Zhu et al. (4) in which full pulpotomy provided larger reductions in postoperative pain levels from day one to day seven, with significantly less pain in day one compared to RCT. Similar magnitude of pain reduction for both pulpotomy and RCT was revealed in other studies (19 , 20); Patel et al. (19) found that both pulpotomy and RCT groups had similar magnitude of pain reduction. Mean pain scores area under curve (AUC) for RCT versus pulpotomy were 245.2 versus 221.55; concluding that pulpotomy was as effective as RCT in reducing postoperative pain. Sar et al. (20) observed that in moderate pulpitis patients, no significant difference between full pulpotomy and RCT in pain scores at any time point evaluated, however, in severe pulpitis patients, a significant difference was found at 24 and 72 hours with higher pain scores in RCT. A systematic review in 2023 by Alhilou et al. (23) highlighted that there were controversies within the available randomized control trials on deciding which treatment (pulpotomy/pulpectomy) was more effective in reducing emergency pain. This urges research to pursue further investigations to solve this dilemma. Variable biomaterials have been reported as pulpotomy capping materials; Mineral Trioxide Aggregate (MTA) is a hydrophilic and biocompatible endodontic cement, capable of stimulating healing and osteogenesis with reported high success rate, it performed better than calcium hydroxide as a pulp capping material in VPT (24). Biodentine (Septodont, St Maur-des-Fosses, France) is a calcium silicate cement which was reported to have high biocompatibility and bioactivity, perfect sealing ability, and high compressive strength. Furthermore, Biodentine was reported to have improved properties compared with MTA; it has shorter setting time and better handling properties. The physical properties of Biodentine were improved by modifying its powder composition and adding accelerator, softener, as well as the replacement of bismuth oxide as a radiopacifier with zirconium oxide for better shade stability (25). Portland cement is a hydraulic binding material widely used in the building industry. It has similar composition to MTA containing tricalcium silicate, dicalcium silicate, tricalcium aluminate, and alumino-ferrite thus, it has been considered a possible alternative pulp capping material. Portland cement differs from MTA by the absence of bismuth ions and the presence of potassium ions (16). Commercial Portland cement has some concerns and drawbacks that can be overcome by applying certain precautions. First, it is not manufactured under sterile conditions; therefore, it must be sterilized for example using via gamma radiation, autoclaving or hot air oven and mixed with sterile water or saline under aseptic conditions. Second, it lacks a radio-opacifier and is not visible on radiographs; thus, it requires mixing with a radio-opacifier like bismuth oxide or zirconium oxide. Third, its arsenic content might be of concern, however, the arsenic levels are low; and the amount released is not a reason for its contraindication (26). Several studies have shown comparable results of Portland cement to MTA in terms of antimicrobial effect (27), induction of cell proliferation, attachment and growth and reparative dentin formation (28 , 29) physical and mechanical properties (30), biocompatibility (31 , 32), as well as the clinical and radiographic effectiveness as a pulp dressing agent in primary molars (33 - 35). Portland cement is inherently much cheaper than MTA due to its widespread industrial availability, therefore Portland cement can provide an economic alternative to MTA as a pulp capping material in emergencies or resource-limited settings with proper precautions (26). However, no previous randomized clinical trial has been carried out to determine the performance of Portland cement as a pulpotomy agent in mature permanent teeth. The success of VPT can be influenced by the level of preoperative pulp inflammation (17 , 18). An objective diagnostic method is testing the level of inflammatory biomarkers associated with tissue degradation through analysis of pulpal blood (36 , 37). Pulp inflammation is a complex protective biological response designed to protect the pulp from injury, while promoting healing and repair. This biological response is a consequence of host-pathogen interactions in which local immune and pulpal cells orchestrate an inflammatory response characterized by vasodilation, release of inflammatory mediators such as transforming growth factor 1 (TGF-1), vascular endothelial cell growth factor (VEGF), C-C chemokine ligand 2 (CCL2), ), CXC chemokine ligand 10 (CXCL10), human beta-defensins (hBDs), interleukins (ILs): IL-1, IL-2, IL-4, IL6, IL-8, IL-10, interferon- (IFN-), tumor necrotic factor- (TNF-), Matrix Metallo-proteinases (MMP-8 and 9) and recruitment of immune cells to the site of injury (38). MMPs are groups of enzymes (endopeptidases) that have been identified with degradation of extra-cellular matrix. They are expressed in normal tissues at low levels but upregulated in inflammation. The level of MMP- 9 levels was significantly elevated in inflamed pulps (39). The assessment of pulpal biomarkers in pulpal inflammation and identification of the threshold between reversible and irreversible pulpitis have been investigated in literature (40 - 44). MMP-9 levels in pulpal blood of asymptomatic patients were significantly lower than patients with reversible and irreversible pulpitis during partial pulpotomy of mature permanent teeth as reported by Mente et al. (43). Ballal et al. (37) collected pulpal fluid to assess MMP-9 level. After 1 year, the inflammatory state of the pulp tissue, as reflected by MMP-9 levels, had a significant impact on pulp survival and likelihood of subsequent failure. Another study by Sharma et al. (44) concluded that the pulpal blood concentration of MMP-9 in teeth with symptomatic irreversible pulpitis, was significantly associated with the outcome of pulpotomy, where it might be used as a potential prognostic biomarker. A recent study by Gerihan et al. (17) revealed that MMP-2 and 9 expression levels were significantly elevated in specimens with irreversible pulpitis compared to reversible pulpitis, while no significant difference was found between the MMP-8 expression level. A recent systematic review and meta-analysis by Karrar et al. (42) reported that low-quality evidence suggested IL-6 and IL-8 demonstrated an adequate level of diagnostic accuracy. Therefore, the present randomized clinical trial was designed with the primary objective to compare postoperative pain after Biodentine and Portland cement full pulpotomy in mature molars with symptomatic pulpitis. Furthermore, it aimed to correlate between the pulpal blood level of MMP-9 as a marker of the state of pulp inflammation with preoperative pain scoring, intra-operative bleeding time during pulpotomy hoping that it might offer a guiding tool for diagnosis of pulp status.

## Materials and Methods

**Figure 1:**
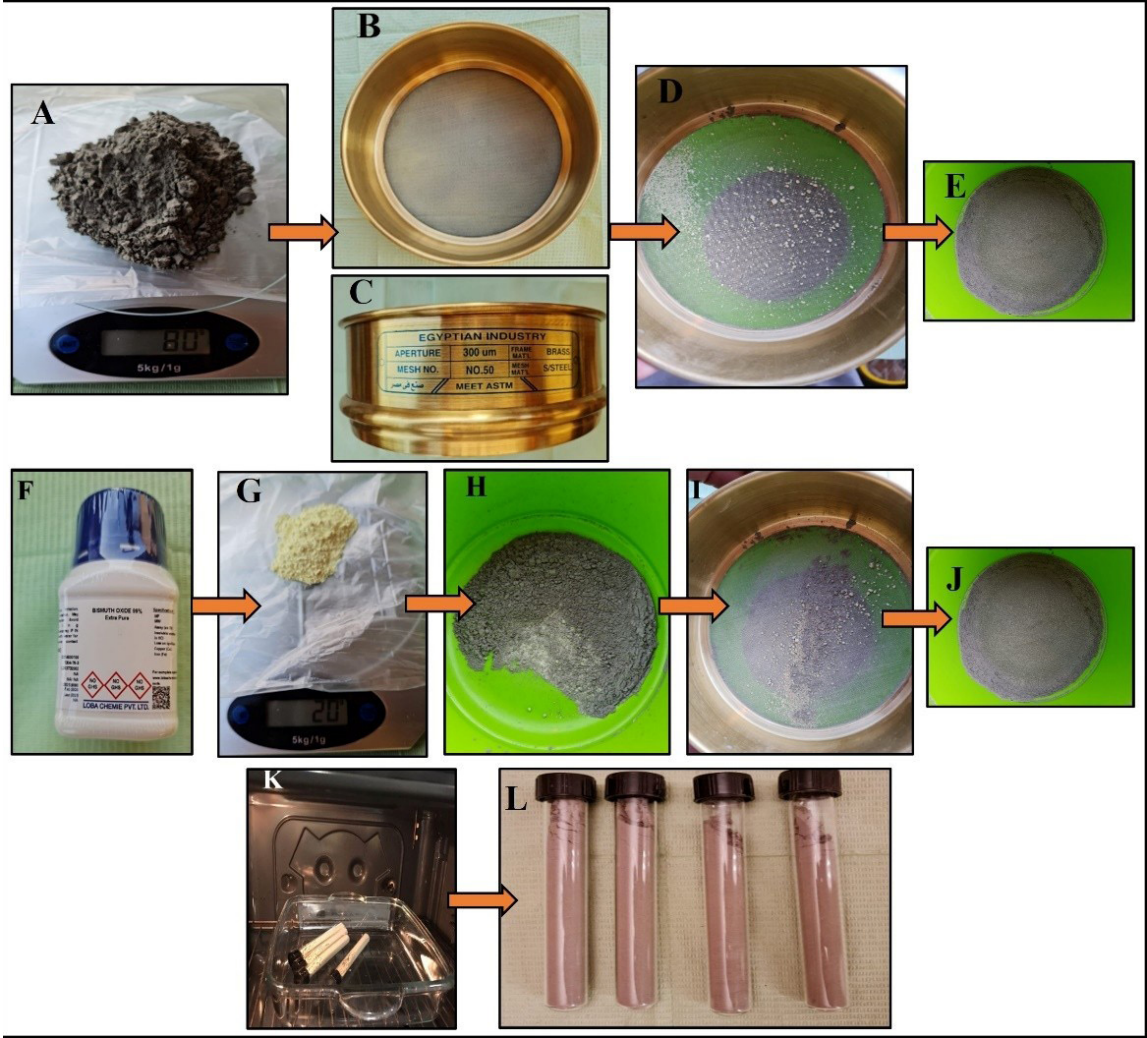
Preparation of Portland cement for dental use: A) 80 grams of industrial grey Portland cement, B,C) 300 µm sieve, D,E) Grey Portland cement passed through a 300 µm sieve to eliminate large particles, F,G) Twenty grams of Bismuth oxide radio-opacifier were weighed, H,I) Adding Bismuth oxide radio-opacifier to the grey Portland cement and sieving them together to properly mix powder particles, J) Final Portland cement/Bismuth oxide powder mixture K) Placement in heat resistant glass tubes and sterilization in a hot air oven of 170 °C for 1 hour, L) Storage in heat resistant glass tubes.

The study design was a two-arm, parallel 1:1 allocation ratio randomized clinical trial. It was designed in accordance with the Consolidated Standards of Reporting Trials (CONSORT) guidelines. The protocol was approved by the Department of Endodontics, Evidence-based Committee and Ethical Committee, Faculty of Dentistry. The approval code of Ethical committee was 46920. The study protocol was registered on www.clinicaltrials.gov (NCT04573374). Guided by Sar et al. (2024) (20), a power analysis was conducted based on an expected difference of 24% between the standard and the tested biomaterial. With a confidence interval of 95%, a probability of a type I error of 5%, and a power at 80%, the sample size was set at 72 participants (36 in each group). Sample size calculation was achieved using PS: Power and Sample Size Calculation Software Version 3.1.2 (Vanderbilt University, Nashville, Tennessee, USA) Participants were recruited from the post-graduate endodontic clinic of the Department of Endodontics, Faculty of Dentistry. The eligibility criteria were patients aged from 18 to 40 years guided by previous studies (15 , 20). Participants had deep occlusal or proximal caries in mature permanent molars with symptomatic pulpitis. The preoperative pain included mild, moderate, or severe pain categories based on the assessment of Numeric Rating Scale (NRS) scores. Bleeding pulp tissue after access cavity confirmed that pulp was vital. The exclusion criteria were medically compromised patients and cases with necrotic pulp and periapical involvement. Exclusion included also: Internal, external root resorption or root canal calcification on radiograph, non-restorable teeth or teeth indicated for post and core restoration, and teeth with severe periodontal disease. Patients who used long-acting non-steroidal anti-inflammatory drugs (NSAIDs) before treatment were excluded as well. A written consent was obtained from the participants before the treatment; where the nature of the treatment, possible side effects, and treatment alternatives were thoroughly clarified. The consent obligated the participant to record a pain diary accurately and to come at follow-up intervals. A detailed medical and dental histories were taken followed by a thorough clinical and radiographic examination. The chief complaint revealed spontaneous pain or pain exacerbated by cold stimuli and lasted for a few seconds or it might be a lingering pain. The patient was asked to mark the level of experienced preoperative pain on NRS. This pain scale consists of a horizontal line with an eleven-point numeric range. It is labelled from zero to ten in which zero represents "No pain" and ten represents "the worst pain possible". The patients were asked to choose the mark that represents their level of pain. Afterwards, pain level was assigned to one of four categories: None (0), Mild (1-3), Moderate (4-6) or Severe (7-10) (45). The NRS is a valid, reliable and easy to understand scale for the measurement of pain intensity when properly explained and applied and was used in previous studies (4 , 14 , 46). Visual examination detected the presence of odontogenic causes such as extensive caries or large restoration. The tooth was initially assessed by thermal stimulation. A Cold test was performed using cold spray (Coltene Roeko Endo-Frost Dental Pulp Tester, Switzerland) which was done by applying cold-sprayed cotton on the middle third of the buccal surface of the tested tooth compared with the contra-lateral tooth and the duration of pain was recorded in seconds. In addition, Electric Pulp Tester (Denjoy DY310 Dental Pulp Tester, Denjoy, Henan, China) was used to record the response of the affected tooth and the control tooth. The affected tooth provided an earlier positive response compared to the contralateral tooth. Percussion and palpation evaluation were negative thus excluding periapical involvement. Preoperative radiographic assessments checked the pulp space morphology, the extent and location of caries as well as the periapical status. Radiographs were taken by an X-ray machine (Bel-Ray, Belmont, Japan) using digital imaging plate (DIGORA Optime digital photostimulable phosphor imaging plate-size 2, Finland). The preoperative radiograph was taken using a standardized paralleling technique in which a bite record was taken using rubber base impression material (Zhermack, Zetaplus C-silicone, Poland) mounted on the Rinn XCP alignment system (Rinn corporation, Elgin, IL) for standardization for future comparison. Tooth type, carious site (occlusal or proximal), presence or absence of pulp exposure, type of caries (primary or secondary), and number of missing walls were all recorded. A total of 72 patients were randomly allocated in 2 groups, each composed of 36 patients. The random sequence was generated by the main supervisor (http://www.random.org/). Local anesthesia of the tooth was achieved using 4% Articaine with 1: 100000 epinephrine (Septodont. Saint-Maur-des-Fosses. France). In mandibular molars, if no lip numbness occurred or numbness occurred with pain during caries removal, another inferior alveolar nerve block injection of the same formulation was administered. The tooth was isolated with a rubber dam, and the crown was disinfected before exposure using cotton pellet soaked with 5% Sodium hypochlorite (FMCG manufacturer, 10th of Ramadan City, Egypt). Carious dentin was removed using a sterile, sharp # 5 round bur round with adequate water cooling. Pulp exposure was done using a new sterile bur. After exposure, pulp bleeding was examined under magnification (Seiler Alpha Air 3 Dental Operating Microscope) and the criteria of bleeding color (light or dark) and profuseness (profuse or minimal) were recorded. Deroofing was completed using Endo Z bur (Endo-Z Bur, DENTSPLY, Tulsa Dental, DENTSPLY Maillefer, TN, USA). For assessment of the level of pulpal blood marker MMP-9, a 10 µL pulpal blood sample was taken using 75 µL heparinized micro-capillary tube (Henso Medical (Hangzhou) Co.,Ltd, Hangzhou, China) placed in the middle of the pulp chamber and transferred immediately to a numbered Eppendorf cup containing 0.75 ml sterile physiological saline solution (Sodium Chloride 0.9%, Febco, Egypt). The numbered Eppendorf cups were stored in a cool box containing cool packs of -25°C until being sent to the laboratory. The assessment of MMP-9 was done using MMP-9 ELISA test (INOVA, Bioneovan Co, Beijing, China). Coronal pulp tissue was amputated using a sharp spoon excavator to the level of canal orifices and the pulp chamber was irrigated with saline solution to remove any pulp traces. Bleeding from canals and attachment of tissues were examined under dental operating microscope magnification and recorded (attached or detached). To achieve hemostasis, a sterile cotton pellet soaked with 2.5% NaOCl was left over the orifices for 2 minutes and repeated 4 times up to 8 minutes (47), if bleeding continued, the process was repeated for another 8 minutes, then for a third set of 8 minutes, if necessary. The total time to control bleeding was recorded. After hemostasis, the pulp chamber was flushed with distilled water. The randomization sequence was kept in 8-fold paper in opaque sealed envelopes. The operator opened the envelope after hemostasis to determine the pulpotomy agent to be placed over the exposed pulp. In the intervention group, teeth were treated with full pulpotomy using Portland cement (ASEC Helwan cement, Egypt), and in the control group, teeth were treated with full pulpotomy using Biodentine (Septodont, St Maur-des-Fosses, France). To prepare Portland cement for dental use, eighty grams of industrial grey Portland cement were weighed on a digital sensitive balance and passed through a 300 µm sieve to eliminate the large particles (48). Twenty grams of Bismuth oxide (99% Extra Pure, LOBA CHEMIE, India) radio-opacifier were weighed and added to the grey Portland cement and sieved together to properly mix the powder particles (48 - 50). The final mix was then put in heat- resistant glass tubes to be sterilized in a hot air oven of 170 °C for 1 hour (51) and stored in the same glass tubes (Fig. 1).

1

**Figure 2:**
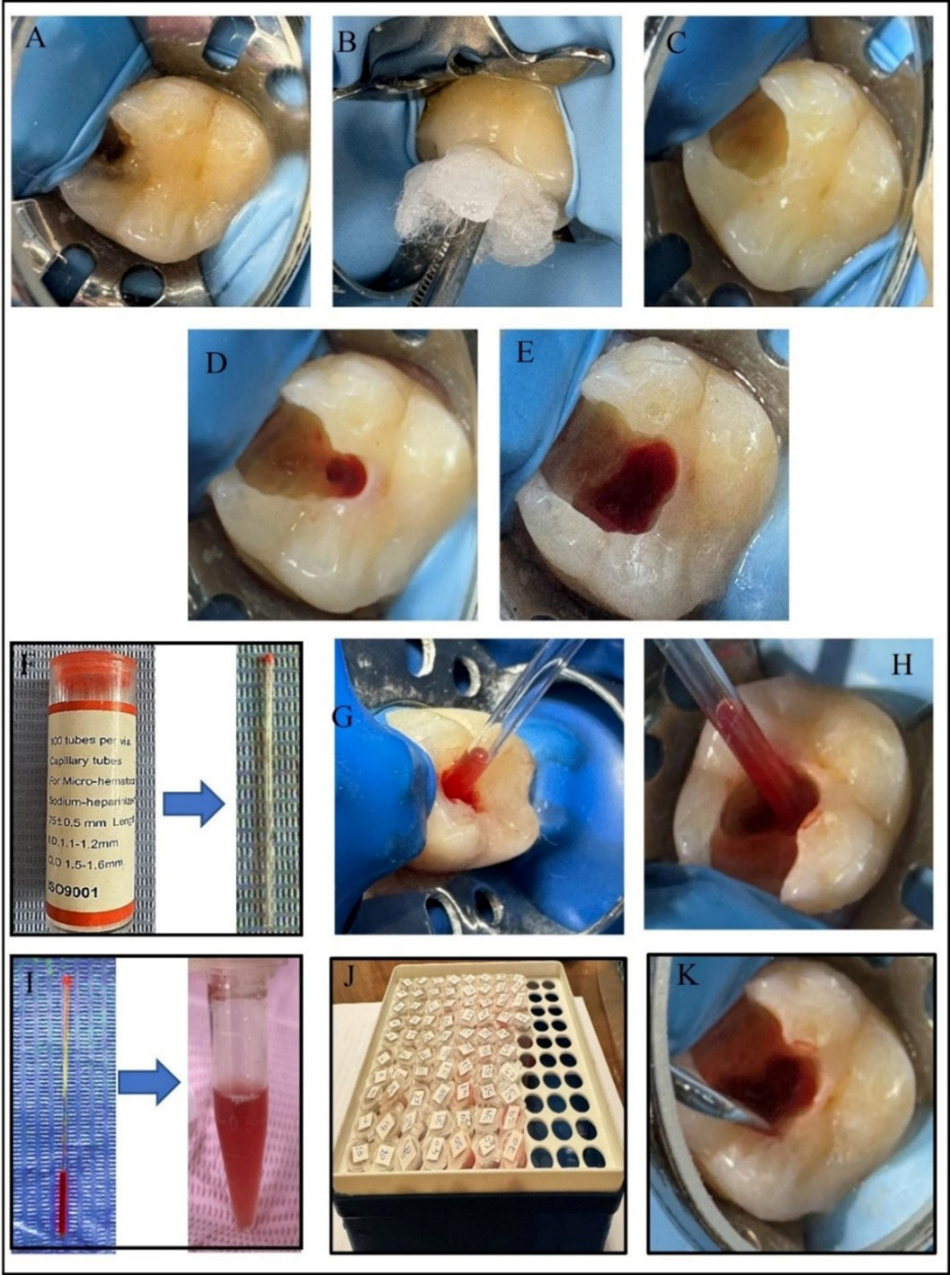
Procedural steps of pulpal blood sampling during full pulpotomy: A, B) Tooth isolation and crown disinfection using cotton pellet soaked with 5% NaOCl, C) Carious dentin removal using sterile, sharp #5 round bur with adequate coolant, D)Pulp exposure, E) Complete deroofing and pulp bleeding examination under DOM, F)Heparinized micro-capillary tube before collecting the sample, G, H )Taking pulpal blood sample using heparinized microcapillary tube placed in the pulp chamber, I) Heparinized micro-capillary tube after collecting the sample then sample transferred to Eppendorf cups containing 0.75 ml sterile saline, J)The collected blood samples in numbered Eppendorf cups, K) Coronal pulp tissue amputation using a sharp spoon excavator to the level of canal orifices.

**Figure 3:**
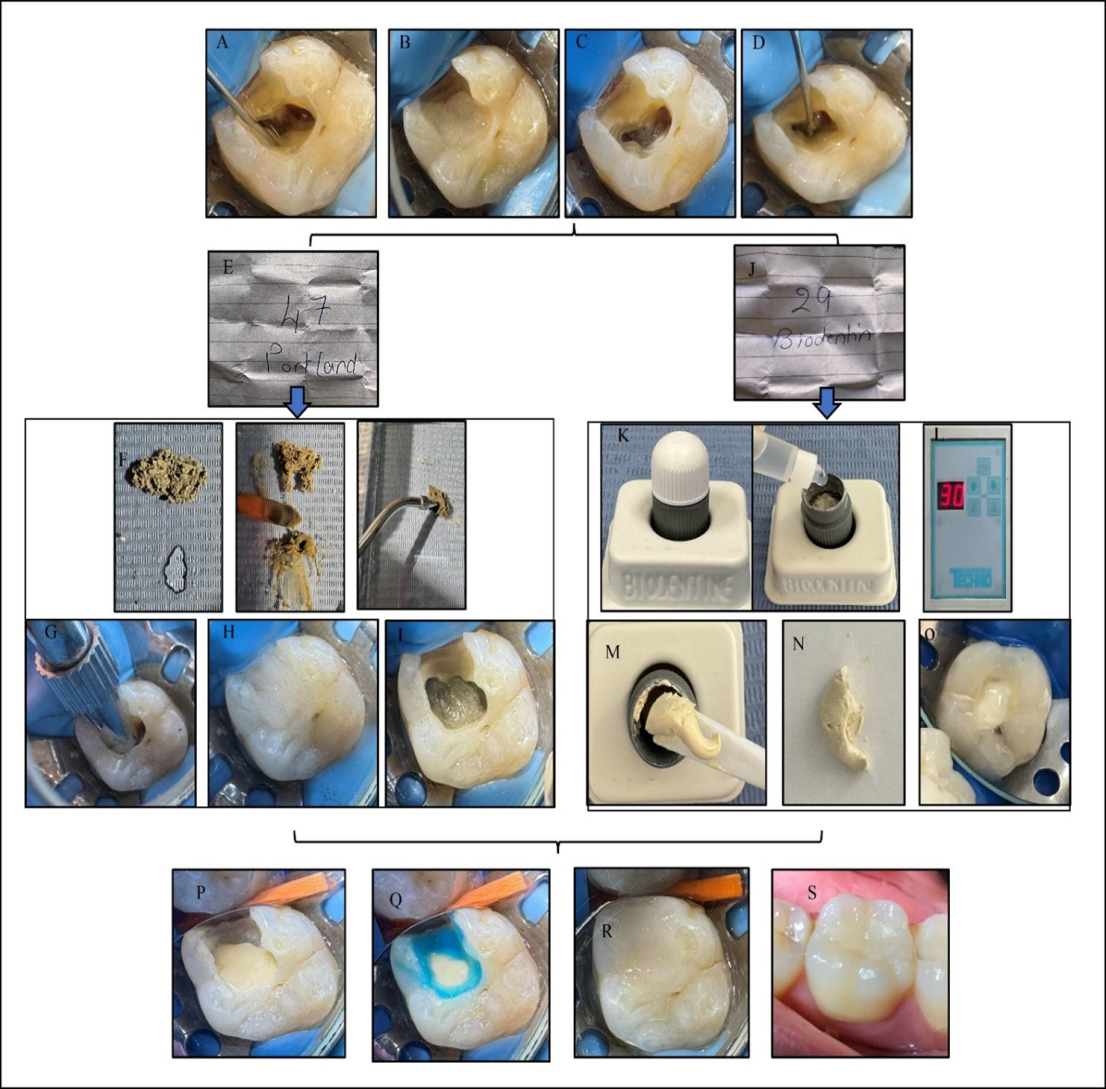
Procedural steps of hemostasis and application of pulpotomy material during full pulpotomy A) pulp chamber irrigation with saline solution to remove any pulp traces, B) Bleeding control with a sterile cotton pellet soaked with 2.5% sodium hypochlorite, C) Pulp stumps after achieving hemostasis, D) Flushing the pulp with distilled water, E) Opening the envelope after hemostasis to determine the pulpotomy agent; Portland cement , F) Portland cement mixing in a 3:1 powder: distilled water ratio with a spatula on a glass slab, G) Placement of Portland cement in 2-3 mm layer using amalgam carrier and condensed, H,I) A cotton pellet moistened with saline was placed in the pulp chamber against Portland cement for 5 seconds to ensure water uptake then removed, J) Opening the envelope after hemostasis to determine the pulpotomy agent; Biodentine K) Dispensing five drops of liquid from the single-dose pipette into the powder-filled capsule placed in the provided stand, L) 30-second trituration in a mixing device at 4,000 vibrations per minute, M,N) The resulting consistency should be a creamy, line-like material along the capsule when opened, Biodentine was collected using a flat plastic instrument, O) Placement of Biodentine in 2-3 mm layer, P) Resin modified glass ionomer placed as a base to seal the pulp chamber, Q) Selective acid etching, R,S) Resin composite final restoration.

In the intervention group, Portland cement powder was mixed with distilled water in a 3:1 powder: distilled water ratio using a glass slab and a metal spatula to reach a workable paste-like consistency. The mix was placed in the pulp chamber using an amalgam carrier to reach a 2-3 mm layer and condensed. A cotton pellet moistened with saline was placed in the pulp chamber against Portland cement for 5 seconds to ensure water uptake, then it was removed; as performed by Petrou et al. (34). In the control group, Biodentine was mixed according to manufacturer's instructions then the mix was collected using a flat plastic instrument, placed in a 2-3 mm layer above the pulp tissue and gently packed using a condenser. Initial setting was achieved after 12 minutes according to the manufacturer. For both groups, a resin modified glass ionomer (EQUIA Forte HT Fil, GC corporation, Tokyo, Japan) was placed as a base to seal pulp chamber. The cavity was restored with resin composite (FILTEK Z350 XT Universal Restorative, 3M ESPE, USA, Bonding system: ALL BOND UNIVERSAL light cured dental adhesive, BISCO, Schaumburg, USA) as an immediate permanent restoration. Figures 2 and 3 show the procedural steps of full pulpotomy and blood sampling.

2

3

**Figure 4:**
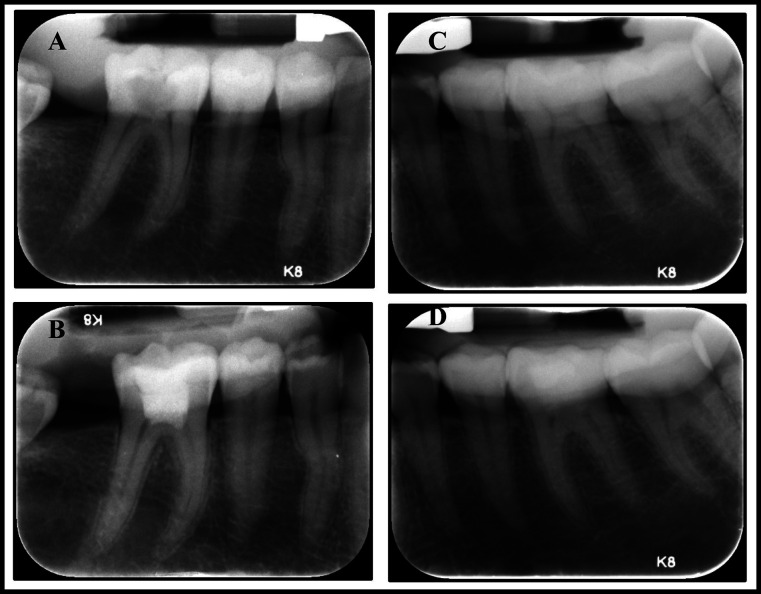
Preoperative and postoperative Radiographs of mandibular first molars treated with full pulpotomy A) Preoperative of case assigned for Portland cement pulpotomy, B) Postoperative after Portland cement full pulpotomy, C) Preoperative of case assigned for Biodentine pulpotomy, D) Postoperative after Biodentine full pulpotomy.

Postoperative periapical radiograph was taken using paralleling technique. The patient's bite record was mounted on the Rinn XCP alignment system, with the same exposure parameters as the preoperative radiograph, (Fig. 4).

4

Postoperative pain records on NRS were taken at the scheduled follow-up periods; 6, 12, 24, 48 hours and 1 week postoperatively. Patients were contacted by telephone by the same operator to remind the patient to record pain score in the pain diary which was delivered after 1 week. The participants were instructed in case of the presence of unbearable postoperative pain to take one tablet of Ibuprofen 600 every 8 hours and to report the number of tablets consumed. In this study the patients were blind to the intervention. The main investigator knew the pulpotomy agent because every material has its own presentation, color and handling properties. Statistical analysis was performed with SPSS 27®, Graph Pad Prism® and Microsoft Excel 2016. All data were tested for normality using the Shapiro-Wilk and Kolmogorov-Smirnov tests which revealed that age and preoperative NRS scores originated from normal data. Comparison between different groups was performed by using Independent t-test. Regarding bleeding time, post operative pain score, number of tablets; data was non-parametric, accordingly comparison between groups was performed by using Mann Whitney test, while intragroup comparisons were performed by using Friedman test. In categorical data, all comparisons were performed by using Chi square test and Fisher's exact test. The significant level was set to be at P 0.05.

## Results

I. Baseline data and Preoperative NRS score: The baseline demographic and clinical characteristics of patients in the Portland cement group and the Biodentine group are presented in Table 1.

Table 1

The results show that the two groups were generally comparable across most variables, with no statistically significant differences. II. Pulp examination under magnification: Across all evaluated parameters including bleeding time, profuseness, color, and attachment to the canal, no statistically significant differences were observed between the two groups. All treated patients achieved hemostasis within the period of 24 minutes. III. Postoperative pain scores: Postoperative pain scores measured at 6 hours, 12 hours, 24 hours, 48 hours, and 1 week following treatment with Portland Cement and Biodentine are presented in Table 2.

Table 2

Inter-group comparisons revealed no statistically significant differences in pain at any time point while intra-group comparisons demonstrated a significant reduction in pain over time for each group (P&lt;0.0001). IV. Frequency of analgesics in take: Frequency of analgesic intake during the first seven days post-treatment revealed that for intergroup comparison, each day showed no statistically significant differences between the two groups. On day one, 47.2% of Portland Cement patients took analgesics once and 27.8% twice, compared with 63.9% and 16.7%, respectively, in the Biodentine group (p = 0.34). On subsequent days, the majority of patients gradually stopped taking analgesics, with no significant differences between groups. Intra-group comparison showed a progressive decrease in analgesic intake over time for both groups. V. Preoperative concentration of MMP-9 (ng/ml): The MMP-9 concentration in the Portland cement group (mean 19.27 ng/ml, median 17.17 ng/ml, SD 8.77) was almost similar to that in the Biodentine group (mean 18.31 ng/ml, median 17.12 ng/ml, SD 7.37). The ranges also overlap substantially (5.91-43.56 ng/ml vs 8.68-40.16 ng/ml); respectively. The P value of 0.74 confirms that the difference of about 0.96 ng/ml in mean values is not statistically significant, suggesting that both groups performed similarly regarding preoperative MMP-9 concentration. VI. Association between MMP-9 (ng/ml) and carious site (occlusal or proximal), pulp exposure, number of missing walls, profuseness, color, and attachment to canal: Association between MMP-9 (ng/ml) and carious site (occlusal or proximal), pulp exposure, number of missing walls, bleeding profuseness, bleeding color, and attachment of pulp tissue to the canals are presented in Table 3.

Table 3

No significant associations were found in both groups. VII. Correlations between MMP-9 level, preoperative NRS score, bleeding time, and postoperative pain: The correlation analysis examining the relationships between preoperative NRS score, bleeding time with MMP-9 level and postoperative pain measurements at various time points (6h, 12h, 24h, 48h, and 1 week) for both Portland cement and Biodentine groups is presented in table 4.

Table 4

In Portland Cement group: Regarding preoperative NRS score and bleeding time, a strong positive correlation was observed indicating that higher preoperative pain scores were associated with longer bleeding times. Preoperative NRS scores as well as bleeding time showed significant positive correlations with postoperative pain measurements at all time points particularly presenting strong correlations with pain at 24 and 48 hours. MMP-9 demonstrated strong positive correlations with both preoperative NRS scores and bleeding time, suggesting that higher MMP-9 levels were associated with greater initial pain intensity and prolonged bleeding duration. In Biodentine group: Similarly, preoperative NRS score and bleeding time showed a strong positive correlation. Preoperative NRS score as well as bleeding time showed significant positive correlation with postoperative pain measurements at all time points. The correlation patterns were comparable to Portland cement group, with all pain measurements at different time points. Interestingly, pain at 6 hours showed a highly strong correlation with NRS score (r=0.918) which was higher than in Portland cement group (r=0.478). MMP-9 also showed strong positive correlations with both NRS scores and bleeding time, indicating similar associations between inflammatory biomarkers, pain, and bleeding as observed in Portland cement group.

## Discussion

In recent years, VPT has gained importance and is practiced as an alternative to RCT in mature permanent teeth with symptomatic pulpitis with the growing evidence of high rates of clinical and radiographic success (3 - 15). This concept was proven successful since 1998 when Nosrat and Nosrat (52) performed VPT procedure on six permanent molars (four adolescents, 10-15 years of age, and two adults, 20 and 27 years of age) with deep carious lesions and pulpal involvement. After achieving hemostasis, they covered the exposed pulp with calcium hydroxide paste followed by Zinc oxide and Eugenol, and semi-permanent restoration. All teeth demonstrated a hard tissue bridge formation within three months and were asymptomatic. They suggested VPT could be an alternative treatment for pulpal exposure of deep carious lesions. Since the main challenge with VPT is the preoperative condition of the pulp, all included participants had teeth initially symptomatic in accordance with previous studies (4 , 6 - 10 , 13 , 14 , 47). Full coronal pulpotomy was the intervention performed in this study to ensure complete removal of inflamed coronal pulp; it also provides firm sheath of the floor of the pulp chamber for proper placement and compaction of the biocompatible capping material. In the process of full pulpotomy pulp bleeding must be controlled to be able to clinically identify potential inflamed tissues that require removal before application of an appropriate biomaterial. To achieve hemostasis, a cotton pellet soaked with 2.5% NaOCl was used as encouraged in recent guidelines (1 , 2). 2.5% NaOCl was left over the orifice for 2 minutes and repeated 4 times up to 8 minutes (47 , 53). If bleeding continued, the process was repeated for 8 minutes then 8 more minutes, if necessary; based on a study that suggested that hemostasis could be achieved up to 25 minutes (6). Participants who have taken NSAIDs 12 hours before treatment were excluded, to avoid drug interactions that could influence the short-term postoperative records or the level of inflammatory mediators since NSAIDs are known to lower MMP-9 values (54 , 55). Biodentine was chosen as a comparator in this study. The reported biological and physical advantages of Biodentine include: being hydrophilic, biocompatible, bioactive material capable of stimulating healing and osteogenesis in addition to excellent sealing properties, adequate mechanical properties and ability to set in moisture and blood contaminated environment (25). Most important is the reported success rates as a pulpotomy capping material in VPT was high ranging from 87-100 % (9 - 11 , 13 , 53). On the other hand, failure rates for Biodentine when used as a pulp capping material were not significantly different from MTA as reported in a recent systematic review and meta-analysis in 2024 by Komora et al. (56). Failure rate odds ratios were 2.26, 2.53 and 2.46 for MTA versus 1.09, 1.21 and 1.47 for Biodentine at 6, 12 and 24 months; respectively. Portland cement contains the same principle chemical elements as MTA, with similar mechanisms of action and physical properties and biocompatibility (26 - 32). Furthermore, several studies have reported that the beneficial effects of MTA are also found in Portland cement in terms of antimicrobial effect (27), induction of cell proliferation, attachment and growth and reparative dentin formation (28 , 29) physical and mechanical properties (30), biocompatibility (31 , 32). Portland cement was proven successful as a pulp dressing agent in randomized clinical trials including only primary molars (33 - 35). However, no randomized clinical trials with regards to management of permanent teeth with Portland cement full pulpotomy were performed; in fact, its performance was only assessed in case reports (57 , 58). In a case report by Albatal et al. (57), after performing a full pulpotomy procedure using Portland cement in a permanent molar with closed apices and irreversible pulpitis, the observations showed complete disappearance of the signs and symptoms after one day. At 6-month follow-up the case showed clinical and radiographic success. Throughout both the diagnosis and the treatment, the patient's pain intensity was measured using the Wong-Baker Faces Pain Rating Scale. They concluded that permanent tooth pulpotomy with Portland cement could help preserve pulp vitality and promote healing. Another case report by Abed et al. (58), pulpotomy was performed on two immature mandibular first permanent molars with irreversible pulpitis using Portland cement. They attributed the possible reasons for the success of one pulpotomy procedure and the failure of another after 2-year follow-up to having the failed tooth restored as a class II cavity that made it more susceptible to microleakage. Thus, it seemed worth performing a randomized clinical trial to assess the performance of Portland cement as a pulp capping material in mature permanent teeth with symptomatic pulpitis. Moreover, considering the low cost and apparently similar properties of Portland cement in comparison to MTA, it is reasonable to hope that Portland cement can be considered as a possible substitute for MTA in endodontic applications. Baseline data were balanced between the two groups; as successful randomization and allocation of participants ensured similar distribution of all factors that may affect the study results. Included patients had symptomatic pulpitis with comparable mean of preoperative NRS scores of 6.58 ± 2.22 and 6.63 ± 1.96 in Portland cement and Biodentine groups; respectively. To confirm the vital diseased pulp condition, intra-operative assessment of pulpal bleeding, tissue color and attachment were performed. In case pulp exposure did not elicit bleeding, the coronal pulp may be infected and necrotic, contraindicating a VPT procedure (2 , 47). The pulp features upon magnification were examined as recommended by recent guidelines (1 , 2). Bleeding profuseness (minimal versus profuse), attachment of tissues to canal walls (attached versus detached) and bleeding color (dark versus light) indicated probable severity of pulp inflammation. The present study did not reveal a significant difference regarding bleeding profuseness, attachment of tissues to canal walls and bleeding color, denoting standardized preoperative pulp condition in both groups. In a study by Aminabadi et al. (59), significant difference in pulpal blood color was found between the different levels of pulpal inflammation that was clinically perceptible by the human eye indicating that blood color could be a valid clinical diagnostic criterion of pulpal status. Postoperative pain or sensitivity might occur after full pulpotomy (2 , 10). It could result due to several causes including incomplete removal of inflamed coronal pulp, remaining inflammation in the radicular pulp, occlusal trauma by high restoration, poor coronal seal or delayed final restoration that allows bacterial ingress leading to ongoing inflammation and pain in addition to chemical or thermal irritation from the capping material (12 , 60). Fortunately, in most cases postoperative pain after full pulpotomy is a transient self-limiting finding that resolves within the first few days after treatment and well responding to analgesics (10). The following intervals were used to assess intensity of postoperative pain, 6 hours, 12 hours, 24 hours, 48 hours and 1 week. These intervals were chosen as 6 hours postoperatively was considered enough time to allow the disappearance of the anesthetic solution's effect (61). However, 12 and 24 hours were chosen because the acute inflammatory response would begin within few hours or days after treatment (61 , 62). For 48 hours, it was chosen since the prevalence and severity of pain substantially decrease within the first 2 days (62). 1 week interval was chosen as most previous studies used this time interval to assess postoperative pain (8 , 19 , 20 , 46 , 63). It was noted that postoperative pain after pulpotomy could last for 1 week after treatment as by day 7, inflammation decreases, macrophages remove necrotic debris, fibroblasts proliferate and pain subsides (64). Regarding the results of the current study, when comparing Portland cement and Biodentine, postoperative pain scores and categories showed insignificant differences at any time point throughout the observation period. Due to the scarcity of studies with regards to management of permanent teeth with Portland cement full pulpotomy, direct comparison of Portland cement and Biodentine in literature was not feasible. However, some comparisons regarding postoperative pain in studies using Biodentine or MTA will be discussed. Regarding Biodentine full pulpotomy, the current study showed mean NRS scores preoperatively, at 24 hours, and at 1 week to be 6.65 (moderate), 3.81 (moderate), and 1.58 (mild); respectively. The trend of postoperative pain decrease was almost similar to the following studies that compared postoperative pain after Biodentine full pulpotomy and RCT (19 , 63). Patel et al. (19) found the average Visual Analogue Scale (VAS) scores for preoperative pain, at 24 hours, and 1 week to be 50 (moderate), 37.3 (mild) and 19.2 (mild); respectively. Similarly, in a study by Taha et al. (63), the mean NRS scores preoperatively, at 24 hours, and 1 week were 7.53 (severe), 3.03 (moderate) and 0.23 (mild); respectively. The difference in pain categories might be due to the difference in the pain scales used. Regarding Portland cement full pulpotomy, the current study showed mean NRS scores preoperatively, at 24 hours and 1 week of 6.58 (moderate), 3.86 (moderate) and 1.78 (mild); respectively. Regarding MTA full pulpotomy, similar trend of postoperative pain decrease over time was observed in studies comparing postoperative pain after MTA full pulpotomy and RCT (8 , 20 , 46). Galani et al. (8) reported that mean 10-cm VAS scores preoperatively, at 24 hours and 1 week were 3.8 (moderate), 1.52 (mild) and 0.0 (pain-free); respectively. Sari et al. (20) reported that the mean 10-cm VAS scores preoperatively, at 24 hours and 1 week in moderate pulpitis group were 5.43 (moderate), 1.65 (mild) and 0.14 (mild); respectively, while in severe pulpitis, the scores were 8.48 (severe), 1.58 (mild) and 0.08 (mild); respectively. Eghbal et al. (46) found the mean NRS scores preoperatively, at 24 hours and 1 week to be 4 (moderate), 1.25 (mild) and 0.3 (mild); respectively. Of interest, postoperative pain results of MTA full pulpotomy were lower when compared to Portland cement. The lower preoperative pain scores might in part account for their lower pain levels. It was noted that the mean preoperative NRS score in the Portland cement group was 6.58 ± 2.22 and decreased to 6.39 ± 3.04 at 6 hours, while in the Biodentine group there was a slight increase in the mean pain score from 6.65 ± 1.96 to 7.33 ± 2.29 at 6 hours, though statistically insignificant. However, the correlation, as represented by Pearson's correlation coefficient (r), changed from moderate (r=0.478) in the Portland cement group to strong (r=0.918) in the Biodentine group. This may be due to flare-up in cases with severe preoperative pain scores that corresponded to this severe pain scores at 6 hours. Pain after Biodentine pulpotomy could be as result of high initial pH, which can cause temporary irritation before healing begins (65). The initial pH of Biodentine is approximately 11-12 and gradually decreases after setting to around 9-10. This alkalinity comes from the release of calcium hydroxide during hydration of tricalcium silicate (66 , 67). The highly alkaline environment causes superficial coagulation necrosis at the pulp-material interface. This irritation stimulates pulpal nociceptors and induces an inflammatory response in the form of vasodilatation and release of inflammatory mediators (e.g., prostaglandins). pH-related pain is usually dull aching, pressure pain starting within the first 24-48 hours and usually self-limiting (1-3 days) (65). The initial pH of MTA is 10-11 (68), which is slightly less than that of Biodentine. This might contribute to less initial pain tendency with MTA. A significant decrease in the proportion of patients reporting severe pain in successive time points in both groups was observed; in the Portland Cement group, the proportion of patients reporting severe pain decreased from 55.6% at 6 hours to only 2.8% at 1 week. The Biodentine group exhibited a similar trend, with severe pain decreasing from 75.0% at 6 hours to 8.3% at 1 week. These trends confirm that using both materials resulted in progressive pain relief over time. The results followed other studies that showed significant reduction in associated pain following full pulpotomy (4 , 8 , 15 , 19 , 20 , 22 , 46 , 63). Regarding MTA, Sari et al. (20) reported severe pain decreasing from 25% at 6 hours to 10% at 12 hours then, 0% at 24 hours after MTA full pulpotomy. Galani et al. (8) reported that most patients had either no or mild pain by the second day postoperatively in the MTA full pulpotomy group. In the current study, frequency of analgesic intake was similar in both groups. Same trend of gradual decrease in analgesic intake was observed in both groups during the first week. This could be due to similar progressive decrease in pain intensity over time in both groups. Taha et al. (63) reported that 20% of patients took analgesics after Biodentine full pulpotomy. Eghbal et al. (46) reported 62.2 % of patients required analgesics after MTA full pulpotomy whereas Galani et al. (8) reported none of the patients in the MTA full pulpotomy group had taken analgesics within 7 days of treatment. Regarding the correlations between the preoperative and the postoperative pain scores, significant positive correlations were observed at all intervals in both groups particularly presenting strong correlations at 24 and 48 hours. Preoperative was reported to be a predictive factor of postoperative pain (69 , 70). A strong significant positive correlation was demonstrated between the concentration of MMP-9 and the preoperative NRS score in both groups suggesting that higher MMP-9 levels were associated with greater initial pain intensity. MMP-9 levels were found to be significantly elevated in inflamed pulps (39). Mente et al. (43) reported that MMP-9 levels in pulpal blood of asymptomatic patients were significantly lower than patients with reversible and irreversible pulpitis. Another study by Sharma et al. (44) reported significant difference between the concentrations of MMP-9 in pulpal blood of teeth with normal pulps and symptomatic irreversible pulpitis. Gerihan et al. (17) revealed that MMP-2 and 9 expression levels were significantly elevated in specimens with irreversible pulpitis compared to reversible pulpitis. Similarly, the results of the current study indicated that the level of MMP-9 in the pulpal blood is a valuable tool to assess the degree of inflammation and destruction of the pulp tissue reflected by the preoperative pain score. By analyzing the scatter diagrams (Figure 5 A-F) representing the correlations between preoperative NRS and concentration of MMP-9 in both groups, it could be said that the level of MMP-9 in the mild pain category is probably (10 ng/ml), in the moderate category (10-20 ng/ml) and in the severe category ( 20 ng/ml). Sharma et al. (44) reported a significant difference between the median concentrations of MMP-9 successful and failed cases of MTA pulpotomy. They also identified the concentration of 334.8 ng/ml MMP-9 as a cutoff to predict the outcome of pulpotomy procedure because of its optimal sensitivity and specificity. Still standardized methods and units of biomarker measurement are recommended in future studies. Regarding the correlation between the MMP-9 level and bleeding time, a strong significant positive correlation was observed in Portland cement and Biodentine groups with r = 0.931 and 0.888; respectively. These findings agreed with the findings of Abdelaziz et al. (18) that showed that bleeding time had a significant moderate positive correlation with molecular findings of MMP-9. This could be attributed to the severity of pulp inflammation reflected by bleeding time and MMP-9 level. In the current study, a strong significant positive correlation was observed between preoperative NRS score and bleeding time in both groups, indicating that higher pain scores were associated with longer bleeding times. Bleeding time could reflect the state of inflammation. Severe bleeding in an inflamed pulp occurs due to vascular and inflammatory changes within the pulp including: the release of inflammatory mediators e.g., histamine, prostaglandins, and bradykinin that lead to marked dilation of pulpal blood vessels and result in increased blood flow and profuse bleeding. Moreover, increased vascular permeability makes bleeding continuous and difficult to control. In addition, inflamed pulp lacks normal vasoconstrictive control and finally, the increased intra-pulpal pressure that leads to capillary rupture and copious bleeding (64). The mean bleeding time revealed no significant difference between the two groups with mean bleeding time of 5.39 ± 3.34 minutes in Portland cement group and 7.11 ± 5.42 minutes in Biodentine group. The minimum bleeding time was 2 minutes, and the maximum bleeding time reached was 16 minutes which was observed in severe pain category. None of the participants reached the suggested period of 24 minutes to achieve hemostasis. None of the previous trials analyzed the mean time to achieve hemostasis except for Taha et al. (47) who recommended that if hemostasis could not be achieved in 4 minutes, further tissue removal was required with a further attempt to achieve hemostasis. Interestingly, bleeding time also showed a significant positive correlation with postoperative pain measurements at all time points in both groups. This suggested that bleeding time, reflecting the degree of inflammation, could be an indicator for short-term postoperative outcome. Insignificant association was found in both groups between the concentration of MMP-9 and caries site (occlusal or proximal), presence or absence of pulp exposure, number of missing walls, profuseness of bleeding, bleeding color, and attachment of tissues to the canals. A previous study by Abdelaziz et al. (18) evaluated the impact of the number of missing walls and MMP-9 level in pulpal blood on the success of full pulpotomy. They found that number of missing walls did not significantly affect the success rate while the levels of MMP-9 showed a significant correlation with the outcomes. This study has a limitation concerning operator blinding, which was not feasible because each material has distinct presentation, color, and handling properties. However, the patients, who were unaware of their treatment allocation, independently assessed their postoperative pain levels. Long-term follow-up is recommended to further evaluate the clinical and radiographic success of the tested materials. An additional consideration is that industrial Portland cement might present regulatory and safety concerns. Therefore, as performed in our study, it should be refined to standardize particle size and properly sterilized for dental use. Moreover, a radiopacifier should be added to Portland cement to allow radiographic assessment of the material. Arsenic content is also a concern. A literature review on arsenic content in Portland cement (71) reported an average arsenic concentration of 6.8 ppm and levels up to 34.27 mg/kg in grey Portland cement. The authors stated that these levels are generally considered low and unlikely to cause toxic effects; therefore, no absolute contraindication for clinical use has been demonstrated. Portland cement has promising biological properties similar to MTA and may be considered a cost-effective alternative. However, further research is required to confirm its long-term clinical safety and suitability for routine clinical practice.

## Conclusions

To date, assessment of Portland cement as a pulpotomy agent in mature permanent teeth has not been carried out in a randomized clinical trial. Comparable postoperative pain outcome to Biodentine suggested that Portland cement can be used as an economic alternative for expensive calcium silicate cements. MMP-9 level in pulpal blood as well as time to achieve hemostasis in full pulpotomy procedure could offer a guiding tool for diagnosis of pulp status. Bleeding time could also predict the degree of postoperative pain after full pulpotomy.

## Figures and Tables

**Table 1:** Comparison of baseline data and preoperative NRS scores of both groups.

		Group				P value
		Portland cement		Biodentine		
Age: Mean ± SD		29.08	6.88	27.29	7.19	0.27
GenderN (%)	Male	3	8.3%	2	5.3%	0.599
	Female	33	91.7%	34	94.7%	
Tooth typeN (%)	Mandibular First Molar	21	58.4%	23	60.5%	0.558
	Mandibular Second Molar	1	2.8%	2	5.3%	
	Maxillary First Molar	13	36.1%	12	31.6%	
	Maxillary Second Molar	1	2.8%	1	2.6%	
Carious site (occlusal or proximal)N (%)	No	0	0.0%	2	5.3%	0.27
	occlusal	11	30.6%	15	39.5%	
	proximal	23	63.9%	16	47.4%	
	both	1	2.8%	3	7.9%	
	buccal pit	1	2.8%	0	0.0%	
True pulp exposureN (%)	No	12	33.3%	11	31.6%	0.87
	Yes	24	66.7%	25	68.4%	
Type of caries (primary or secondary)N (%)	Primary	29	80.6%	32	89.5%	0.57
	Secondary	2	5.6%	2	5.3%	
	Deep restoration	5	13.9%	2	5.3%	
Number of missing wallsN (%)	No (occlusal)	11	30.6%	16	44.7%	0.44
	1	23	63.9%	18	50.0%	
	2	2	5.6%	2	5.3%	
Preoperative NRS score: Mean ± SD		6.58	2.22	6.63	1.94	0.99
Preoperative NRS CategoryN (%)	Mild	5	13.9%	4	13.2%	0.99
	Moderate	11	30.6%	11	31.6%	
	Severe	20	55.6%	21	55.3%	

*Significant difference as P≤0.05.

**Table 2:** Pain scores at different time points in both groups.

	Group								P value
	Portland cement				Biodentine				
	Min.	Max.	Mean	SD	Min.	Max.	Mean	SD	
Baseline	2.00	10.00	6.58a	2.22	3.00	9.00	6.65 a	1.96	0.99
6h	0.00	10.00	6.39 a	3.04	0.00	10.00	7.33 a	2.29	0.265
12 h	0.00	8.00	4.67 ab	2.48	0.00	9.00	4.56 b	2.81	0.968
24 h	0.00	9.00	3.86 b	2.51	0.00	9.00	3.81 b	2.67	0.954
48 h	0.00	8.00	3.22 bc	2.11	0.00	10.00	3.39 bc	2.82	0.937
1 week	0.00	8.00	1.78 c	2.07	0.00	9.00	1.58 c	2.55	0.277
P value	<0.0001*				<0.0001*				

*Significant difference as P≤0.05.

**Table 3:** Association between MMP-9 (ng/ml) and Carious site (occlusal or proximal), Pulp exposure, Number of missing walls, Profuseness, Color, Attachment to canal in both groups.

			Concentration of MMP-9 (ng/ml)		P value		Concentration of MMP-9 (ng/ml)		P value
		Portland cement	Mean	SD		Biodentine	Mean	SD	
Carious site (occlusal or proximal)	Occlusal		18.55	10.79	0.82		17.41	7.79	0.35
	Proximal		19.62	8.29			18.83	7.44	
Pulp exposure	No		15.08	4.52	0.06		18.37	7.85	0.99
	Yes		21.36	9.67			18.28	7.30	
Type of caries (1ry or 2ry)	Primary		20.06	9.34	0.28		17.57	6.86	0.38
	Secondary		10.98	2.03			24.49	14.45	
	Deep restoration		16.60	3.61			23.94	8.92	
Number of missing walls	0 (occlusal)		16.16	6.92	0.25		18.43	7.94	0.13
	1		21.13	9.47			19.03	7.04	
	2		14.99	4.17			10.61	0.93	
Bleeding profuseness	Profuse		19.63	9.23	0.92		17.98	7.25	0.82
	Minimal		18.77	8.37			18.89	7.86	
Bleeding color	Light		18.77	8.37	0.92		18.89	7.86	0.83
	Dark		19.63	9.23			17.98	7.25	
Attachment to canal	Detached		18.68	8.35	0.69		16.92	5.66	0.38
	Attached		20.01	9.49			20.25	9.13	

3

**Table 4:** Correlation between bleeding time. Pain and MMP-9 (ng/ml) in both groups.

Correlations	Portland Cement				Biodentine			
	Preoperative NRS Score		Bleeding Time (min)		Preoperative NRS Score		Bleeding Time (min)	
	r	P value	r	P value	r	P value	r	P value
Preoperative NRS Score			0.773	0.0001*			0.788	0.0001*
Bleeding Time (min)	0.773	0.0001*			0.788	0.0001*		
Pain after 6h	0.478	0.0001*	0.644	0.0001*	0.918	0.0001*	0.643	0.0001*
Pain after 12 h	0.518	0.0001*	0.436	0.0001*	0.622	0.0001*	0.259	0.0001*
Pain after 24 h	0.717	0.0001*	0.842	0.0001*	0.701	0.0001*	0.836	0.0001*
Pain after 48 h	0.726	0.0001*	0.894	0.0001*	0.727	0.0001*	0.896	0.0001*
Pain after 1 week	0.619	0.0001*	0.285	0.0001*	0.551	0.0001*	0.637	0.0001*
MMP-9 (ng/ml)	0.776	0.0001*	0.931	0.0001*	0.778	0.0001*	0.888	0.0001*

*Significant difference as P≤0.05.

## Data Availability

The datasets used and/or analyzed during the current study are available from the corresponding author.
